# Alternate Day Fasting Improves Endothelial Function in Type 2 Diabetic Mice: Role of Adipose-Derived Hormones

**DOI:** 10.3389/fcvm.2022.925080

**Published:** 2022-05-26

**Authors:** Jian Cui, Sewon Lee, Yan Sun, Cuihua Zhang, Michael A. Hill, Yuhang Li, Hanrui Zhang

**Affiliations:** ^1^School of Traditional Chinese Medicine, Beijing University of Chinese Medicine, Beijing, China; ^2^Dalton Cardiovascular Research Center, School of Medicine, University of Missouri, Columbia, MO, United States; ^3^Department of Medical Pharmacology and Physiology, School of Medicine, University of Missouri, Columbia, MO, United States; ^4^Division of Sport Science, College of Arts and Physical Education, Incheon National University, Incheon, South Korea; ^5^Department of Medicine, Division of Cardiology, Cardiometabolic Genomics Program, Columbia University Irving Medical Center, New York, NY, United States

**Keywords:** adipose, adipokines, diabetes, diet, endothelial function, alternate day fasting, adiponectin

## Abstract

**Introduction:**

Intermittent fasting, including alternate day fasting (ADF), has grown in popularity as it can produce clinically significant metabolic benefits and is often considered to be easier to adhere to than other types of diets such as chronic calorie restriction. However, the effects of ADF on diabetes-associated vascular dysfunction, and the role of adipose-derived hormones, i.e., adipokines, in mediating its effects, remain largely unknown.

**Objective:**

We aimed to test the hypothesis that ADF protects against diabetes-associated endothelial dysfunction, at least partly through modulating adipokine profiles.

**Methods:**

Control mice (*m Lepr*^db^**) and diabetic mice (*Lepr^db^*) were treated with 12-weeks of ADF. Glucose metabolism, endothelial function, and adipokine profile were assessed.

**Results:**

ADF reduced fasting blood glucose level and homeostatic model assessment for insulin resistance (HOMA-IR), and improved insulin sensitivity. ADF improved endothelium-dependent vasorelaxation of small mesenteric arteries (SMA) of *Lepr^db^* mice. The improvement in endothelial function was largely attenuated by incubation with the nitric oxide synthase inhibitor, L-NAME. These ADF-induced metabolic and vascular benefits were accompanied by increased circulating adiponectin. Adenovirus-mediated adiponectin supplementation improved endothelial function in *Lepr^db^* mice, supporting endothelial protective roles in diabetes-associated endothelial dysfunction. Protein tyrosine nitration is a post-translational modification that serves as a marker of oxidative stress. Nitrotyrosine protein levels in SMA and mesenteric adipose tissue (MAT) were elevated in *Lepr^db^* mice. ADF reduced nitrotyrosine protein in SMA, but not in MAT, of *Lepr^db^* mice.

**Conclusion:**

ADF exerts metabolic and endothelial protective benefits. The improvement of endothelial function was partly mediated by increased adiponectin, representing an important mechanism for the beneficial vascular effects resulting from ADF.

## Introduction

Obesity and diabetes are associated with an increased risk of cardiovascular diseases, which remain the leading cause of death globally (1). Lifestyle modification improves metabolic syndrome and associated systemic inflammation and cardiovascular dysfunction (1). Dietary intervention represents an effective lifestyle modification strategy in the management of obesity and type 2 diabetes (2, 3). Calorie restriction, a form of dietary intervention that involves a reduced daily caloric intake, exerts physiological benefits with extended longevity and decreased risks for many age-related diseases in model organisms and humans (3). Despite the well-known benefits, long-term energy restriction is unlikely to be a feasible lifestyle modification strategy in humans due to poor sustained adherence (3).

Intermittent fasting has grown in popularity in the past few years (4–7). Multiple clinical trials support that short-term intermittent fasting can produce clinically significant metabolic benefits in obese subjects (5). Compared with traditional forms of dietary intervention such as chronic calorie restriction that generally require individuals to vigilantly monitor energy intake, intermittent fasting is often considered to be easier in regard to adherence (8). An intermittent fasting regimen can be defined as periods of fasting alternating with periods of eating. Some of the most studied intermittent fasting methods include alternate day fasting (ADF) (0–500 kcal per fast day alternating with *ad libitum* intake on feast days), the 5:2 fasting (two fast days and five feast days per week), and time-restricted fasting (eating only within a prescribed window of time each day) (5).

Despite the recent surge in popularity, there are limited clinical studies on the effects of long-term intermittent energy restriction in patients with type 2 diabetes (5). Encouragingly, a recent randomized clinical trial in subjects with type 2 diabetes compared an intermittent energy restriction diet (the participants followed 500–600 kcal per day for 2 non-consecutive days per week while following their usual diet for the other 5 days) or a continuous energy restriction diet (1200–1500 kcal per day followed for 7 days per week) for 12 months. The results supported that intermittent energy restriction is an effective alternative strategy for the reduction of hemoglobin A1c (HbA1c) and is comparable to continuous energy restriction in these patients (9).

With the promising results, further clinical and experimental studies are required to explore the benefits and mechanisms of intermittent fasting in type 2 diabetes and associated cardiovascular complications. Mice that are homozygous for the diabetes spontaneous mutation (*Lepr^db^*) have been widely used to model type 2 diabetes (10, 11). Indeed, experimental studies in *Lepr^db^* mice support that intermittent fasting improved glycemic control, lipid dysregulation (12, 13), hepatic steatosis (14), retinopathy (15), and cognitive impairment (16). However, if and how intermittent fasting may rescue vascular dysfunction in type 2 diabetes has not been studied. Furthermore, adipose-derived hormones, i.e., adipokines, have been implicated to be altered in type 2 diabetes and contribute to impaired endothelial function (17–19). Adiponectin is an adipocyte-derived hormone that increases with weight loss and protects the endothelium by decreasing oxidative stress and inflammation (20–23). Leptin and resistin, in contrast, are adipokines that are positively correlated to body weight and fat mass (24), and weight loss decreased their levels (25). Leptin and resistin have been implicated to cause endothelial dysfunction by promoting oxidative stress (17, 19). In view of this, it is plausible to speculate that weight loss strategies, such as ADF, would increase adiponectin and decrease leptin and resistin concentrations. In turn, the improvements in adipokine profiles may have a protective effect on the vasculature resulting in improved endothelial function.

Here we aimed to determine the effects of ADF on glucose metabolism and endothelial function, and the involvement of adipokines, in type 2 diabetic mice and their respective non-diabetic controls. We hypothesized that ADF regulates the adipokine profile, particularly adiponectin, which exerts vascular protective effects in type 2 diabetic mice with ADF. To test this hypothesis, heterozygote control mice (*m Lepr*^db^**) and homozygote type 2 diabetic mice (*Lepr^db^*) were treated with ADF for 12 weeks. We determined: (1) if and how ADF affects weight loss, glucose metabolism, and insulin sensitivity in type 2 diabetic mice; (2) if and how ADF affects circulating adipokines and their expression in adipose tissue; (3) how ADF rescues endothelial dysfunction, and if and how adipokines, including adiponectin, resistin, and leptin, modulated by ADF may contribute to the vascular benefits of ADF; and (4) whether and to what extent ADF influences glucose metabolism and vascular function in non-diabetic control mice.

## Materials and Methods

### Animal Models and Treatment

The procedures followed were in accordance with approved guidelines set by the Animal Care Committee at the University of Missouri (Columbia, MO, United States). Mice were purchased from the Jackson Laboratory (Bar Harbor, ME, United States). Homozygote type 2 diabetic mice (*Lepr^db^*; BKS.Cg-*Dock7*^m^** + / + *Lepr^db^*J, JAX 000642, black and obese) are wildtype for *Dock7* and homozygous for *Lepr^db^* (10, 11). The heterozygote control mice (*m Lepr*^db^**, black and lean) from the colony are heterozygous for *Dock7^m^* and heterozygous for *Lepr^db^*. Because of the sterility of *Lepr^db^* homozygotes, the misty (*Dock7^m^*) mutation has been incorporated into stocks for maintenance of the diabetes mutation (10, 11). The repulsion double heterozygote facilitates the identification of heterozygotes for breeding (10, 11). Mice that are homozygous for *Dock7^m^* and wildtype for *Lepr* from the colony (gray and lean) are discarded (10, 11). The *Lepr^db^* mice demonstrate morbid obesity, chronic hyperglycemia, and are polyphagic, polydipsic, and polyuric (10, 11). Heterozygotes control mice (*m Lepr^db^*) are normal in body weight, blood glucose, and plasma insulin (10, 11).

Mice were maintained on a standard rodent diet. Twelve to sixteen week-old, male, 20–35 g *m Lepr*^db^**, and 40–60 g *Lepr^db^* mice were used in this study. Mice were fed *ad libitum* on alternate days and then moved to a separate cage without food (starting at 9 a.m.) for 24 h to prevent food from being stored in the bedding. Mice were maintained on a 12-hour light/dark cycle and between 18–23°C according to animal protocols and NIH guidelines. To determine the effects of recombinant resistin treatment, *Lepr^db^* mice were treated with murine recombinant resistin (Biovision, Cat# 4560-100, 15 μg/per mouse/day, i.p. injection, for 4 days) as previously described (26).

### Measurement of Body Weight and Abdominal Girth

Body weight was determined using an electronic balance. Abdominal girth was measured with the use of a soft ruler (27).

### Measurement of Blood Parameters

After animals were anesthetized with pentobarbital sodium (50 mg/kg i.p.), whole blood samples were obtained from the vena cava. A whole blood sample was held for 30 min at room temperature to allow clotting. The sample was centrifuged at 2,000–3,000 *g* for 10 min at 4°C; the serum was transferred to separate tubes without disturbing blood clots and stored at −80°C until analysis. Commercial ELISA kits were used to measure serum levels of adiponectin (Millipore, EZMADP-60K), resistin (R&D, MRSN00), and leptin (Millipore, EZML-82K) (28). Similarly, total cholesterol levels were assessed using spectrophotometric assays (Biovision, K603-100) according to the manufacturer’s instructions (28).

### Measurement of Homeostatic Model Assessment for Insulin Resistance

Fasting blood glucose levels were measured by OneTouch Ultramini glucometer (LifeScan). Fasting serum insulin level was measured with the use of a commercial kit (ALPCO, 80-INSMSU-E01). Insulin resistance was determined by the homeostatic model assessment for insulin resistance; homeostatic model assessment for insulin resistance (HOMA-IR) using the following formula: HOMA-IR = (glucose [mmol/L]) × (insulin [mU/L])/22.5 (21).

### Insulin Tolerance Test

Mice were fasted overnight and weighed. The tail was nicked with a fresh razor blade by horizontal cut of the tip and a OneTouch Ultramini glucometer was used to measure baseline blood glucose after overnight fasting. 1.0 unit per kg body weight of diluted porcine insulin (Sigma-Aldrich, St. Louis, MO, United States, I5523) was subsequently injected into the intraperitoneal cavity. Blood glucose was sampled from the tail of each mouse by gently massaging a small drop of blood onto the glucometer strip at 0 (baseline), 15, 30, 60, and 90 min following insulin injection (29).

### Adenovirus-Mediated Gene Transfer

Adenovirus vector containing the gene for full-length mouse adiponectin (Ad-APN) was generously gifted by Dr. Shinji Kihara (Department of Internal Medicine and Molecular Science, University of Osaka, Osaka, Japan) (30). HEK-293 AD cells were passaged two to three times before infection and cultured until the floating monolayer was 90–100% confluent. Cells were replenished with 15 ml new growth media containing 10% FBS per 75-cm^2^ flask with either Ad-APN or adenovirus containing β-galactosidase (Ad-βgal) added to the culture. After 24 h, 10 ml growth media was added to the culture flask, and the viruses were allowed to produce for another 24 h. When all cells were floating, the culture flask was gently shaken, and all media, including the cells, were transferred to a 50-ml sterile tube, which was centrifuged at 1,000 *g* for 5 min. The cell pellet was then resuspended in 0.8 ml culture medium. The adenoviruses were released from the cells by repeated freeze-thaw cycles using liquid nitrogen. After centrifugation at 10,000 *g* for 10 min, the supernatant was collected for immediate purification of adenoviruses using a commercial purification kit (Cell Biolabs, San Diego, CA, United States). The concentration of purified adenovirus was measured using the commercial QuickTiter Adenovirus Titer Immunoassay Kit (Cell Biolabs, San Diego, CA, United States) according to the manufacturer’s instructions. Either Ad-APN or Ad-βgal at 1 × 10^9^ plaque-forming units was injected intravenously *via* the tail vein of *m Lepr*^db^** and *Lepr^db^* mice. All mice were euthanized 7 days after virus injection. Mice that did not show an increase in serum adiponectin levels (as measured by ELISA) were excluded from the functional assessment (20).

### Functional Assessment of Small Mesenteric Arteries

Mesenteric arteries (first order of main branches) with internal diameter of 200–250 μm were cut into 2 mm long rings and mounted in a Myograph 610M (ADInstruments, Colorado Springs, CO, United States) (31). The passive tension-internal circumference was determined by stretching to achieve an internal circumference equivalent to 60–70% of that of the blood vessel under a transmural pressure of 100 mmHg. Vessels were maintained in Physiological Saline Solution (PSS) bubbled with 95% O_2_–5% CO_2_ at 37°C for the remainder of the experiment. PSS contained 118.99 mM NaCl, 4.69 mM KCl, 1.18 mM KH_2_PO4, 1.17 mM MgSO_4_•7H_2_O, 2.50 mM CaCl_2_•2H_2_O, 14.9 mM NaHCO_3_, 5.5 mM D-Glucose, and 0.03 mM EDTA. After an equilibration period of 45 min, vessels were precontracted with 1 μmol/L phenylephrine (PE). A cumulative dose–response curve was obtained by adding acetylcholine (ACh, 1 nmol/L–10 μmol/L) and sodium nitroprusside (SNP, 1 nmol/L–10 μmol/L). Relaxation at each concentration was measured and expressed as the percentage of force generated in response to 1 μmol/L PE. NO availability was evaluated by ACh concentration-response curve repeated after incubation with the NO synthase inhibitor N-Nitro-L-arginine methyl ester (L-NAME, 100 μM, 20 min). PE-induced vasoconstriction was evaluated by cumulative addition of PE (1 nmol/L to 10 μmol/L). The contraction induced by PE was normalized to the maximal force of contraction induced by 120 mM of KCl (27, 32).

### Determination of mRNA Expression by Real-Time Polymerase Chain Reaction

Total RNA was extracted from 10 mg of mesenteric adipose tissue (MAT) using RNeasy Lipid Tissue Mini Kit (Qiagen, Valencia, CA, United States) (27, 32). The quality and quantity of total RNA were determined using a Nanodrop ND-1000 Spectrophotometer (Nano Drop Technologies, Wilmington, DE). Total RNA (1.0 μg) was processed directly to cDNA synthesis using SuperScript™ III Reverse Transcriptase (Invitrogen, Grand Island, NY, United States). Quantitative real-time PCR analyses were performed using an i-Cycler (I-Q5, Bio-Rad Laboratories, Hercules, CA, United States). Reactions were carried out in triplicate in a total volume of 25 μl using SYBR green qPCR Master Mix (Invitrogen, Grand Island, NY, United States). The 2^–ΔΔC^_T_ method (–ΔΔC_T_ = C_T,target gene_ – C_T,Actb_, where C_T_ is threshold cycle) was used to analyze the change of target gene expression. The housekeeping gene *Actb* was used for internal normalization. Mean C_T_ values for both the target and internal control genes were determined. Results are presented as fold change of transcripts for target normalized to internal reference (*Actb*), compared with *m Lepr*^db^** (defined as 1.0 fold) (27, 32). The primer sets were designed by Primer3 (33): mouse *Adipoq* (forward primer: 5′-AGGTTGGATGGCAGGC-3′ and reverse primer: 5′-GTCTCACCCTTAGGACCAAGAA-3′), *Retn* (forward primer: 5′- TTCCTTGTCCCTGAACTGCT-3′ and reverse primer: 5′-CAAGACTGCTGTGCCTTCTG-3′), *Lep* (forward primer: 5′- TGTCCAGGGTTGATCTCACA-3′and reverse primer: 5′- TCCCACTGGAACAAAACTCC-3′), and *Actb* (forward primer: 5′-GCTCTTTTCCAGCCTTCCTT-3′ and reverse primer: 5′-CTTCTGCATCCTGTCAGCAA-3′) (20). The efficiency of the PCR reaction was determined using a dilution series of a standard MAT sample.

### Protein Expression by Western Blot Analyses

In total, 10 mg of MAT, or 4-6 branches of small mesenteric arteries (SMA) were homogenized in lysis buffer (Cellytic™ MT Mammalian Tissue Lysis/Extraction Reagent, Sigma-Aldrich, St. Louis, MO, United States). Protein concentration was assessed using BCA™ Protein Assay Kit (ThermoScientific, Rockford, IL, United States), and equal amounts of protein were separated by SDS-PAGE and transferred to PVDF membranes (Bio-Rad, Hercules, CA, United States). Protein expression was detected using anti-nitrotyrosine primary antibody (Abcam, Cat#ab7048, 1:500) anti-tubulin primary antibody (Abcam, Cat#ab6160, 1:5,000), Horseradish peroxidase-conjugated secondary antibodies were used. Signals were visualized by enhanced chemiluminescence (Santa Cruz Biotechnology, Santa Cruz, CA, United States), scanned densitometrically using Fuji LAS3000 and quantified with Multigauge software (Fujifilm). The relative amounts of protein expression were quantified to those of the corresponding *m Lepr*^db^** control, which was set to a value of 1.0 (27, 32).

### Data Analysis

All data were presented as mean ± SEM except as specifically stated. For insulin tolerance tests and vasomotor responses under various dosages, two-way repeated ANOVA was used to determine how the responses were affected by two factors (ADF × Time as the repeated measure for insulin tolerance tests, and ADF × dosage as the repeated measure for vasomotor responses), followed by the *post hoc* Tukey’s test for multiple comparisons. For other data obtained from the four groups (*m Lepr*^db^**, *m Lepr*^db^** + ADF, *Lepr^db^*, *Lepr^db^* + ADF), one-way ANOVA was used to compare the groups defined by one factor, followed by the *post hoc* Fisher’s Least Significant Difference test for multiple comparisons. Statistical analyses were performed using SPSS11.5. Significance was accepted at *P* < 0.05. The sample size was empirically determined based on our previous studies examining the metabolic and endothelial function of *Lepr^db^* mice and their respective *m Lepr*^db^** control mice (20, 27, 28, 32, 34, 35). Mice were grouped by genotype and weight-matched thus not randomized. Blinded analysis was not performed because of feasibility.

## Results

### The Effects of Alternate Day Fasting on Body Weight, Abdominal Girth, Fasting Total Cholesterol, Fasting Glucose, Fasting Insulin, and Homeostatic Model Assessment for Insulin Resistance in Control and Diabetic Mice

Mice on the ADF diet were allowed to eat *ad libitum* for one day, and then given no food the next day. This regimen was maintained for 12 weeks. Meanwhile, mice without ADF were continually allowed to feed *ad libitum*. Food intake was measured on the feast day for mice with or without ADF once per week. ADF led to a net reduction of food intake at 41.8 ± 4.1% in *m Lepr*^db^** mice and 40.2 ± 4.7% in *Lepr^db^* mice. As expected, the *Lepr^db^* diabetic mice showed higher body weight, abdominal girth, total cholesterol, blood glucose, insulin, and HOMA-IR than the *m Lepr*^db^** control mice. In *m Lepr*^db^** control mice, 12-week ADF reduced body weight, abdominal girth, and insulin. Although ADF modestly reduced total cholesterol, glucose, and HOMA-IR in *m Lepr*^db^** control mice, the *P*-values were greater than 0.05, we thus cannot conclude that significant differences exist. In *Lepr^db^* mice, ADF did not significantly reduce body weight, abdominal girth, and total cholesterol. ADF, however, remarkably reduced blood glucose and HOMA-IR without affecting insulin levels (Table 1), supporting the profound effects of ADF on improving glucose metabolism despite the lack of effects on weight loss in diabetic mice.

**TABLE 1:** Basic characteristics.

	*m Lepr*^db^**	*m Lepr*^db^** + ADF	*Lepr^db^*	*Lepr*^db^** + ADF
Body weight, g	30.88 ± 0.51	27.38 ± 0.63*^#^	55.95 ± 1.38*	53.73 ± 0.49*
Abdominal girth, cm	8.68 ± 0.15	8.05 ± 0.21*^#^	12.10 ± 0.07*	12.02 ± 0.07*
Total cholesterol, mg/dl	158.46 ± 24.27	131.73 ± 11.12^#^	213.37 ± 20.15*	173.78 ± 14.87
Blood glucose, mg/dl	183.80 ± 17.98	150.00 ± 7.28^#^	489.60 ± 58.25*	182.25 ± 23.83^#^
Insulin, ng/ml	1.69 ± 0.32	0.29 ± 0.05*^#^	2.94 ± 0.45*	2.86 ± 0.46*
HOMA-IR	21.58 ± 4.69	3.06 ± 0.67^#^	101.12 ± 21.68*	37.45 ± 9.20^#^

Data represent mean ± SEM. n = 6 mice. *P < 0.05 vs. m Lepr^db^ control mice, # p < 0.05 vs. Lepr^db^ diabetic mice.

### Alternate Day Fasting Improved Insulin Sensitivity in Both Control and Diabetic Mice

**FIGURE 1:**
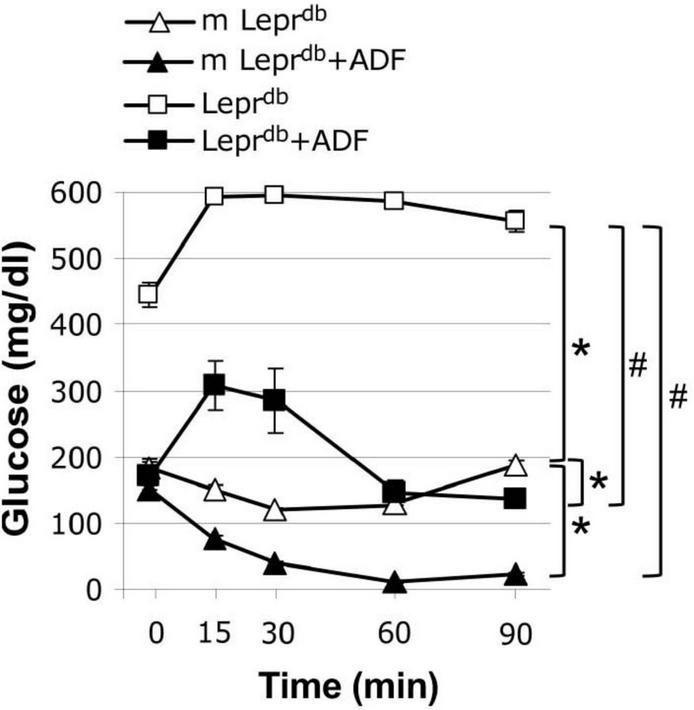
Alternate day fasting (ADF) improved insulin sensitivity in both *m Lepr*^db^** and *Lepr^db^* mice. Insulin tolerance tests revealed that diabetic mice (*Lepr^db^*) had impaired glucose metabolism compared with control mice (*m Lepr*^db^**). Twelve weeks of alternate-day fasting (ADF) improved insulin sensitivity in both *Lepr^db^* and *m Lepr*^db^** mice. Data represent mean ± SEM. *n* = 6 *m Lepr*^db^**, 6 *m Lepr*^db^** + ADF, 6 *Lepr^db^*, and 5 *Lepr^db^* + ADF. *P < 0.05 vs. *m Lepr*^db^** control mice, # *p* < 0.05 vs. *Lepr^db^* diabetic mice.

We further determined how ADF affects insulin sensitivity by performing an insulin tolerance test. As expected, the *Lepr^db^* diabetic mice showed impaired insulin sensitivity. ADF improved insulin sensitivity in both *m Lepr*^db^** control mice and *Lepr^db^* diabetic mice (Figure 1).

### Alternate Day Fasting Rescued Endothelial Dysfunction of Small Mesenteric Arteries in Diabetic Mice

**FIGURE 2:**
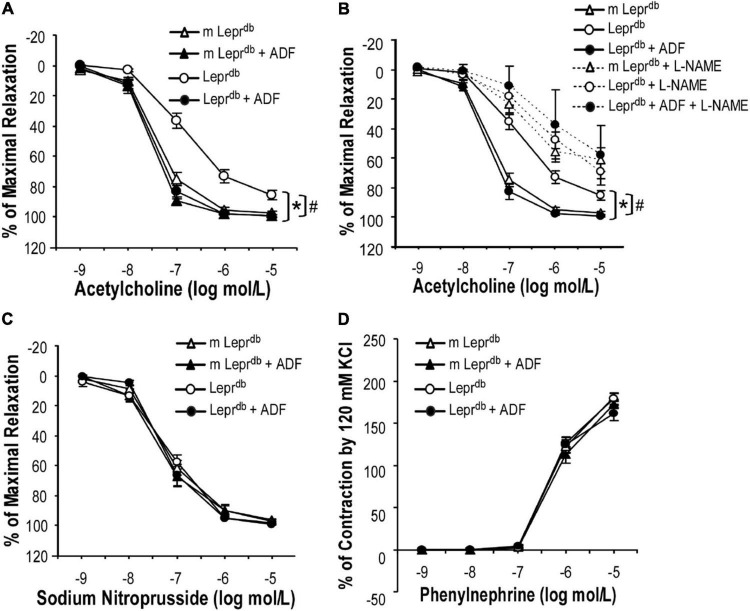
ADF improved endothelium-dependent vasorelaxation without affecting endothelium-independent vasorelaxation or phenylephrine-induced vasoconstriction of small mesenteric arteries (SMA) in type 2 diabetic mice. **(A)** Acetylcholine (ACh)-induced endothelium-dependent vasorelaxation of SMA was impaired in *Lepr^db^* mice. Twelve weeks of ADF in *Lepr^db^* (*Lepr^db^* + ADF) restored endothelium-dependent vasorelaxation back to the level of *m Lepr*^db^** control mice. **(B)** Incubation with the nitric oxide synthase inhibitor (L-NAME) largely attenuated ADF-induced improvement of endothelial function in diabetic mice. **(C)** Sodium Nitroprusside (SNP)-induced endothelium-independent vasorelaxation of SMA was comparable among groups. **(D)** Phenylephrine (PE)-induced vasoconstriction of SMA was comparable among groups. Data represent mean ± SEM. *n* = 5 mice per group with 1–2 rings per mouse. *P < 0.05 vs. *m Lepr*^db^** control mice, # *p* < 0.05 vs. *Lepr^db^* diabetic mice.

SMA represent resistance arteries contributing to vascular resistance and regulation of blood flow. Acetylcholine (ACh)-induced endothelium-dependent vasorelaxation was impaired in SMA of *Lepr^db^* diabetic mice vs. *m Lepr*^db^** control mice. ADF remarkably improved the endothelial function of diabetic mice (Figure 2A). Sodium nitroprusside (SNP)-induced endothelium-independent vasorelaxation (Figure 2C) and phenylephrine (PE)-induced vasoconstriction (Figure 2D) were comparable among groups. Incubation with the nitric oxide synthase inhibitor (L-NAME) largely attenuated the ADF-induced improvement of endothelial function in diabetic mice (Figure 2B). In *m Lepr*^db^** mice, ADF also showed a trend toward increasing the % of maximal relaxation at an ACh concentration of 10^–7^ mol/L. ADF did not affect endothelium-independent vasorelaxation or PE-induced vasoconstriction in *m Lepr*^db^** control mice (Figure 2).

### Alternate Day Fasting Modulates Circulating Adipokine Profile and Their Expression by Adipose Tissue

**FIGURE 3:**
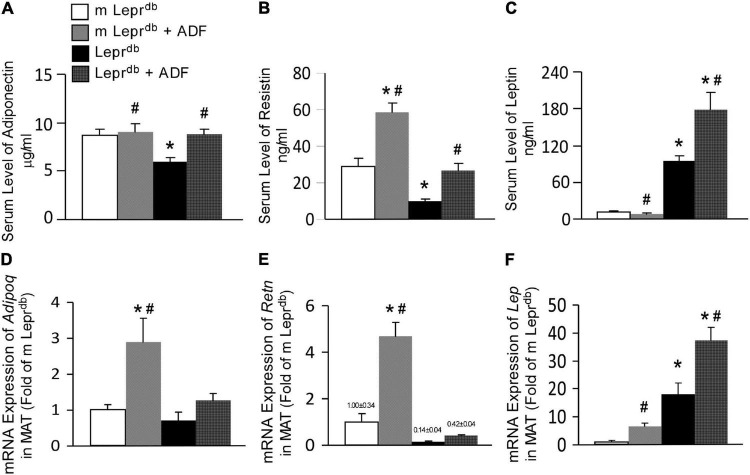
ADF modulated circulating levels and adipose expression of key adipokines, including adiponectin, resistin, and leptin in mesenteric adipose tissue (MAT) of both *m Lepr*^db^** and *Lepr^db^* mice. Circulating levels of adiponectin **(A)**, resistin **(B)**, and leptin **(C)** were determined by ELISA. mRNA expression of *Adipoq* **(D)**, *Retn* **(E)**, and *Lep* **(F)** were determined by qRT-PCR in mesenteric adipose tissue (MAT). Data represent mean ± SEM. *n* = 6 *m Lepr*^db^**, 6 *m Lepr*^db^** + ADF, 6 *Lepr^db^*, and 5 *Lepr^db^* + ADF. *P < 0.05 vs. *m Lepr*^db^** control mice, # *p* < 0.05 vs. *Lepr^db^* diabetic mice.

Adiponectin levels were lower in the serum of *Lepr^db^* mice, and ADF increased serum adiponectin back to the level of *m Lepr*^db^** control mice (Figure 3A). Serum resistin was lower in *Lepr^db^* mice than in *m Lepr*^db^** control mice. ADF increased resistin in both *m Lepr*^db^** control mice and *Lepr^db^* diabetic mice (Figure 3B). As expected, *Lepr^db^* mice showed dramatically increased serum leptin, i.e., hyperleptinemia due to the lack of leptin receptor. ADF reduced serum leptin in *m Lepr*^db^** control mice, yet further increased serum leptin in *Lepr^db^* mice (Figure 3C).

Adipose tissue is the primary source of adipokines released into circulation (36). We further determined mRNA expression of these adipokines in MAT. Consistent with the trend of circulating adipokine levels, *Lepr^db^* mice showed increased *Lep* mRNA (encoding leptin) in MAT (Figure 3F), while reduced *Retn* mRNA (encoding resistin) (Figure 3E) compared with *m Lepr*^db^** control mice. *Adipoq* mRNA (encoding adiponectin) in MAT was not statistically different between *m Lepr*^db^** and *Lepr^db^* mice (Figure 3D). ADF increased *Adipoq* mRNA in MAT of *m Lepr*^db^** control mice, though circulating adiponectin in *m Lepr*^db^** control mice was not altered by ADF (Figure 3D), likely suggesting post-transcriptional regulatory mechanisms limiting a further increase in circulating levels. ADF further increased *Lep* mRNA in the MAT of *m Lepr*^db^** control mice, though serum leptin was reduced by ADF (Figure 3E).

Overall, in *Lepr^db^* mice, adipokine levels in the serum and MAT showed consistent directionality following ADF. In *m Lepr*^db^** control mice, however, the increase in MAT mRNA expression of *Adipoq* and *Lep* did not correlate with change in the circulation, likely suggesting possible feedback mechanisms to maintain homeostasis in non-diabetic control mice.

### The Endothelial Protective Effects of Alternate Day Fasting Were Partly Mediated Through Enhanced Adiponectin

**FIGURE 4:**
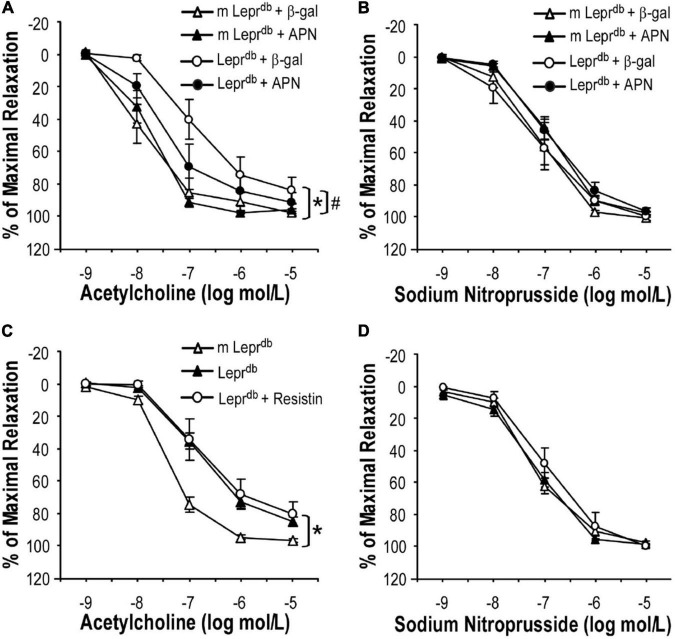
The effects of adipokines on endothelium-dependent vasorelaxation of SMA. **(A)** Adenovirus-mediated adiponectin supplementation improved ACh-induced endothelium-dependent vasorelaxation of SMA in *Lepr^db^* mice, without affecting SNP-induced endothelium-independent vasorelaxation **(B)**. Data represent mean ± SEM. *n* = 5 mice per group with 1–2 rings per mouse. **(C,D)** Treatment with recombinant resistin did not affect endothelium-dependent or endothelium-independent vasorelaxation of SMA. Data represent mean ± SEM. *n* = 6 *m Lepr*^db^**, 6 *Lepr^db^*, 4 *Lepr^db^* + resistin with 1–2 rings per mouse. *P < 0.05 vs. *m Lepr*^db^** control mice, # *p* < 0.05 vs. *Lepr^db^* diabetic mice.

To determine if ADF improved endothelium-dependent vasorelaxation through modulating adipose-derived hormones, we treated control and diabetic mice with adenovirus expressing adiponectin (Ad-APN) or β-galactosidase (Ad-βgal) as the control. As we have previously described (20), mice were euthanized one week after adenovirus treatment and increased circulating adiponectin levels were confirmed by ELISA. Indeed, adiponectin supplementation partly improved endothelium-dependent vasorelaxation in *Lepr^db^* mice (Figure 4A), without affecting endothelium-independent vasorelaxation (Figure 4B). To dissect if other adipokines were also involved in ADF-mediated endothelial protective effects, *Lepr^db^* mice were treated with recombinant murine resistin (15 μg/mouse/day for 4 days, i.p.) according to a previously published protocol (26). The short-term treatment of resistin did not impair or improve endothelium-dependent or -independent vasorelaxation (Figures 4C,D), suggesting that short-term resistin administration may not affect vascular function. Similarly, *Lepr^db^* mice showed increased circulating leptin due to leptin receptor deficiency. Circulating leptin was further enhanced by ADF, suggesting that an increase in circulating leptin itself was unlikely to prevent the vascular benefits of ADF. Thus, our study provided some mechanistic insights into the contribution of adipokines to ADF-mediated vascular effects in type 2 diabetes.

### The Effects of Alternate Day Fasting on the Expression of Nitrotyrosine Protein, a Marker of Oxidative Stress, in Small Mesenteric Arteries and Mesenteric Adipose Tissue

**FIGURE 5:**
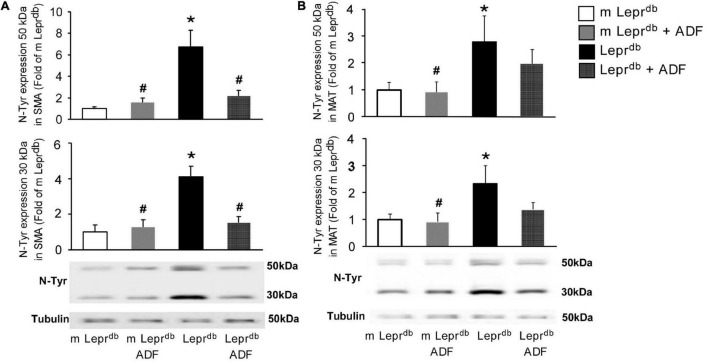
ADF reduced nitrotyrosine protein levels in SMA, but not MAT, of *Lepr^db^* mice. **(A)** Nitrotyrosine protein levels were higher in SMA of *Lepr^db^* mice. ADF reduced nitrotyrosince protein in SMA of *Lepr^db^* mice. *n* = 4 mice per group. **(B)** Nitrotyrosine protein levels were higher in MAT of *Lepr^db^* mice. ADF did not alter nitrotyrosince protein in MAT of *Lepr^db^* mice. *n* = 6 mice per group. *P < 0.05 vs. *m Lepr*^db^** control mice, # *p* < 0.05 vs. *Lepr^db^* diabetic mice.

Nitrotyrosine protein levels were elevated in both SMA and MAT of *Lepr^db^* diabetic mice compared with *m Lepr*^db^** control mice. ADF reduced SMA nitrotyrosine protein levels in *Lepr^db^* diabetic mice without affecting that in the *m Lepr*^db^** control mice (Figure 5A). ADF, however, did not significantly decrease MAT nitrotyrosine protein levels (Figure 5B).

## Discussion

Studies demonstrate that intermittent fasting improves cardiometabolic risk factors such as blood pressure, levels of low-density lipoprotein cholesterol and triglycerides, insulin resistance, and HbA1c (5). A better understanding of how intermittent fasting affects cardiovascular function and the underlying mechanisms will facilitate its clinical application in obesity and diabetes-associated cardiovascular complications. Our study revealed the profound benefits of ADF in rescuing endothelial dysfunction. The benefits are at least partly mediated through enhanced adiponectin, while resistin and leptin were unlikely to be involved. Adiponectin thus provides a mechanistic link between the role of ADF in regulating adipokine profile and endothelial function in type 2 diabetes. ADF reduced the marker of oxidative stress in resistance arteries but not adipose tissue, suggesting tissue-specific regulatory roles by ADF. ADF may also exert metabolic and vascular benefits in non-obese control mice. Overall, our data support that ADF presents as promising lifestyle intervention for treating diabetes-associated endothelial dysfunction.

Intermittent fasting is emerging as a popular alternative dietary intervention strategy. Despite limited numbers of clinical trials directly comparing the long-term effects of intermittent fasting and daily calorie restriction, current evidence supports equivalent or superior metabolic benefits of intermittent fasting (5). Comparative studies in a 12-month study of insulin-resistant participants support that ADF may produce greater reductions in fasting insulin and insulin resistance compared with calorie restriction despite similar decreases in body weight (37). Experimental studies in C57BL/6J mice suggested that ADF produces similar beneficial modulation of body fat distribution and adiponectin levels as does daily calorie restriction (38). In *Lepr^db^* type 2 diabetic mice and streptozotocin-treated type 1 diabetic mice treated with a fasting-mimicking diet, both intermittent fasting and continuous calorie restriction significantly reduced fasting blood glucose levels and improved insulin sensitivity. Yet, intermittent fasting performed significantly better than continuous calorie restriction in improving glycemic control and insulin sensitivity in *Lepr^db^* type 2 diabetic mice (39). Clinical studies, conducted over multiple years, that directly compare different regimens will provide important insights into the long-term cardiometabolic benefits of these diets.

There are currently no clinical studies determining the vascular benefits of long-term ADF in patients with diabetes. Clinical trials of short-term ADF, e.g., 8–12 weeks, have, however, provided promising evidence. In obese subjects (*n* = 32), ADF with a low-fat diet, but not a high-fat diet, for 8-weeks showed improved brachial artery flow-mediated vasodilation (40). Increases in adiponectin were positively associated with augmented flow-mediated vasodilation post-ADF in those subjected to ADF with the low-fat diet (40). ADF also reduced plasma resistin and leptin, which were not correlated with changes in flow-mediated vasodilation (40). In a study involving 54 obese non-diabetic subjects with an 8-week ADF protocol, brachial artery flow-mediated vasodilation was positively correlated to adiponectin concentrations (41). Another study involving 64 obese subjects supported that a 12-week period of ADF improved brachial artery flow-mediated vasodilation (42). Our experimental data strongly support the profound endothelial protective effects of ADF in mice modeling severe type 2 diabetes. To our knowledge, this is the first experimental study determining the role of ADF in diabetes-associated vascular dysfunction. The above clinical studies in obese subjects and our experimental study in type 2 diabetic mice provide premises to further explore the clinical benefits of long-term ADF in diabetes-associated cardiovascular complications.

Our study has shed light on the mechanisms of the endothelial protective effects of ADF partly through enhanced circulating adiponectin. Adiponectin is well known for its anti-inflammatory and anti-oxidative roles in endothelial cells (43) and its protective effects against neointimal formation in response to vascular injury (44) and atherosclerosis (45). Our previous work has also supported that adiponectin abates diabetes-induced endothelial dysfunction by suppressing oxidative stress, adhesion molecules, and inflammation in type 2 diabetic mice (20). Specifically, adenovirus-mediated adiponectin supplementation improved endothelium-dependent vasorelaxation of aortas in *Lepr^db^* mice (20). Adiponectin supplementation reduced aortic nitrotyrosine protein levels, *via* suppressing protein expression of gp91*^phox^*, an NADPH oxidase subunit, and increasing protein expression of SOD3, an antioxidant enzyme (20). Aortic expression of inflammatory genes, *Tnf*, *Il6*, and *Icam1*, was also suppressed by adiponectin supplementation (20). These pathways are likely responsible for the endothelial protective and anti-oxidative effects of adiponectin in mesenteric arteries of *Lepr^db^* mice undergoing ADF. The adiponectin-independent endothelial protective and anti-oxidative effects of ADF remain to be further dissected, and we speculate that the metabolic benefits of ADF may play important roles.

Alternate day fasting exerts profound metabolic benefits in both control and diabetic mice with remarkably improved glycemic control and insulin sensitivity. The effects of ADF on weight loss and visceral adiposity were, however, modest. In particular, ADF led to ∼4% weight loss in *Lepr^db^* mice that did not reach statistical significance (Table 1). Consistent with our observation, an independent study also suggested that a 13-week period of intermittent fasting, using a fasting mimicking diet protocol, improved glucose homeostasis in *Lepr^db^* mice without causing weight loss (39). Our previous study examining the effects of bariatric surgery in *Lepr^db^* mice demonstrated a ∼15–18% weight reduction following gastric bypass surgery that was accompanied by significantly improved glycemic control and insulin sensitivity (27). Thus, the metabolic benefits of ADF in *Lepr^db^* diabetic mice are likely not entirely dependent on weight loss effects. Since the *Lepr^db^* mice resemble severe type 2 diabetes, whether ADF may also exert limited benefits in weight management in patients with type 2 diabetes, despite profound metabolic effects, should be studied clinically. In *m Lepr*^db^** control mice, ADF led to statistically significant weight loss (∼11%, Table 1), and further improved glucose metabolism with a trend toward improved endothelial function. Further, the benefits of ADF in non-obese, healthy humans thus may also warrant further investigation.

There are many questions that remain to be explored. Future studies may further elucidate if the knockout of adiponectin abolishes the vascular protective effects of ADF, the involvement of other adipokines, and the molecular mechanisms by which ADF modulates adipokine expression and secretion. Comparative studies are required to tackle how different intermittent fasting regimens affect metabolic, vascular, and hormonal parameters. Findings generated from such studies could inform whether one regimen is superior to the others and elucidate the mechanisms that underlie the cardiometabolic benefits. The discovery of pharmacological agents mimicking fasting can potentially provide novel therapeutic strategies. A potential limitation of the present studies is that they were performed only in male mice and mesenteric resistance arteries. Given possible sexual dimorphism in regard to obesity/diabetes-related macrovascular and microvascular disease, it will be important to extend our observations to females and in multiple vascular beds, such as aorta and coronary arteries, as well as the microvasculature.

## Conclusion

In summary, our study examined the role and mechanisms of ADF in diabetes-associated endothelial dysfunction using murine models of type 2 diabetes. We have revealed that ADF in type 2 diabetic mice exerts profound endothelial protective effects, partly through modulating the adipose-derived hormone, adiponectin. Thus, this study improves our understanding of how ADF affords significant protection against endothelial dysfunction partly by regulating adipose-derived hormones. Our work also elaborated on the metabolic benefits and potential cardiovascular protective actions of ADF in the management of type 2 diabetes.

## Dedication

The manuscript is in memory of Dr. Cuihua Zhang, who was deceased on October 1, 2011.

## Data Availability Statement

The raw data supporting the conclusions of this article will be made available by the authors, without undue reservation.

## Ethics Statement

The animal study was reviewed and approved by the Animal Care Committee at the University of Missouri (Columbia, MO, United States).

## Author Contributions

JC, HZ, and CZ conceived the study. JC, SL, and HZ performed the experiments. JC and HZ analyzed the data. JC, YL, and HZ interpreted results of experiments and drafted the manuscript. JC, YS, and HZ prepared the tables and figures. JC, SL, YS, MH, YL, and HZ edited and revised the manuscript. All authors contributed to the article and approved the submitted version.

## Conflict of Interest

The authors declare that the research was conducted in the absence of any commercial or financial relationships that could be construed as a potential conflict of interest.

## Publisher’s Note

All claims expressed in this article are solely those of the authors and do not necessarily represent those of their affiliated organizations, or those of the publisher, the editors and the reviewers. Any product that may be evaluated in this article, or claim that may be made by its manufacturer, is not guaranteed or endorsed by the publisher.

## Abbreviations

ACh: acetylcholine
ADF: alternate-day fasting
MAT: mesenteric adipose tissue
NO: nitric oxide
PE: phenylephrine
SMA: small mesenteric artery
SNP: sodium nitroprusside.

## References

[1] Powell-Wiley TM Poirier P Burke LE Després JP Gordon-Larsen P Lavie CJ Obesity and cardiovascular disease: a scientific statement from the american heart association. *Circulation.* (2021) 143:e984–1010. 10.1161/cir.0000000000000973 33882682 PMC8493650

[2] Varady KA Cienfuegos S Ezpeleta M Gabel K. Cardiometabolic benefits of intermittent fasting. *Annu Rev Nutr.* (2021) 41:333–61. 10.1146/annurev-nutr-052020-041327 34633860

[3] Flanagan EW Most J Mey JT Redman LM. Calorie restriction and aging in humans. *Annu Rev Nutr.* (2020) 40:105–33. 10.1146/annurev-nutr-122319-034601 32559388 PMC9042193

[4] Santos HO Genario R Tinsley GM Ribeiro P Carteri RB Coelho-Ravagnani CF A scoping review of intermittent fasting, chronobiology, and metabolism. *Am J Clin Nutr.* (2022) 115:991–1004. 10.1093/ajcn/nqab433 34978321

[5] Varady KA Cienfuegos S Ezpeleta M Gabel K. Clinical application of intermittent fasting for weight loss: progress and future directions. *Nat Rev Endocrinol.* (2022) 18:309–21. 10.1038/s41574-022-00638-x 35194176

[6] Vasim I Majeed CN DeBoer MD. Intermittent fasting and metabolic health. *Nutrients.* (2022) 14:631. 10.3390/nu14030631 35276989 PMC8839325

[7] Zang B-Y He L-X Xue L. Intermittent fasting: potential bridge of obesity and diabetes to health? *Nutrients.* (2022) 14:981. 10.3390/nu14050981 35267959 PMC8912812

[8] Martens CR Seals DR. Practical alternatives to chronic caloric restriction for optimizing vascular function with ageing. *J Physiol.* (2016) 594:7177–95. 10.1113/jp272348 27641062 PMC5157076

[9] Carter S Clifton PM Keogh JB. Effect of intermittent compared with continuous energy restricted diet on glycemic control in patients with type 2 diabetes: a randomized noninferiority trial. *JAMA Netw Open.* (2018) 1:e180756. 10.1001/jamanetworkopen.2018.0756 30646030 PMC6324303

[10] Chen H Charlat O Tartaglia LA Woolf EA Weng X Ellis SJ Evidence that the diabetes gene encodes the leptin receptor: identification of a mutation in the leptin receptor gene in Db/Db mice. *Cell.* (1996) 84:491–5. 10.1016/s0092-8674(00)81294-5 8608603

[11] Chua SC Jr. Chung WK Wu-Peng XS Zhang Y Liu SM Tartaglia L Phenotypes of mouse diabetes and rat fatty due to mutations in the Ob (Leptin) receptor. *Science.* (1996) 271:994–6. 10.1126/science.271.5251.994 8584938

[12] Zhou J Jiang Z Lin Y Li C Liu J Tian M The daily caloric restriction and alternate-day fasting ameliorated lipid dysregulation in type 2 diabetic mice by downregulating hepatic pescadillo 1. *Eur J Nutr.* (2022). 10.1007/s00394-022-02850-x [Epub ahead of print]. 35290477

[13] Zhang H Zhang W Yun D Li L Zhao W Li Y Alternate-day fasting alleviates diabetes-induced glycolipid metabolism disorders: roles of Fgf21 and bile acids. *J Nutr Biochem.* (2020) 83:108403. 10.1016/j.jnutbio.2020.108403 32497958

[14] Kim KE Jung Y Min S Nam M Heo RW Jeon BT Caloric restriction of Db/Db mice reverts hepatic steatosis and body weight with divergent hepatic metabolism. *Sci Rep.* (2016) 6:30111. 10.1038/srep30111 27439777 PMC4954985

[15] Beli E Yan Y Moldovan L Vieira CP Gao R Duan Y Restructuring of the gut microbiome by intermittent fasting prevents retinopathy and prolongs survival in Db/Db mice. *Diabetes.* (2018) 67:1867–79. 10.2337/db18-0158 29712667 PMC6110320

[16] Liu Z Dai X Zhang H Shi R Hui Y Jin X Gut microbiota mediates intermittent-fasting alleviation of diabetes-induced cognitive impairment. *Nat Commun.* (2020) 11:855. 10.1038/s41467-020-14676-4 32071312 PMC7029019

[17] Zhang H Zhang C. Adipose “Talks” to distant organs to regulate insulin sensitivity and vascular function. *Obesity (Silver Spring).* (2010) 18:2071–6. 10.1038/oby.2010.91 20395945 PMC2946982

[18] Zhang H Zhang C. Regulation of microvascular function by adipose tissue in obesity and type 2 diabetes: evidence of an adipose-vascular loop. *Am J Biomed Sci.* (2009) 1:133. 10.5099/aj090200133 20098632 PMC2809393

[19] Zhang H Cui J Zhang C. Emerging role of adipokines as mediators in atherosclerosis. *World J Cardiol.* (2010) 2:370–6. 10.4330/wjc.v2.i11.370 21179304 PMC3006473

[20] Lee S Zhang H Chen J Dellsperger KC Hill MA Zhang C. Adiponectin abates diabetes-induced endothelial dysfunction by suppressing oxidative stress, adhesion molecules, and inflammation in type 2 diabetic mice. *Am J Physiol Heart Circ Physiol.* (2012) 303:H106–15. 10.1152/ajpheart.00110.2012 22561304 PMC3404646

[21] Zhang H Park Y Zhang C. Coronary and aortic endothelial function affected by feedback between adiponectin and tumor necrosis factor α in type 2 diabetic mice. *Arterioscler Thromb Vasc Biol.* (2010) 30:2156–63. 10.1161/atvbaha.110.214700 20814014 PMC2959138

[22] Lee S Park Y Dellsperger KC Zhang C. Exercise training improves endothelial function via adiponectin-dependent and independent pathways in type 2 diabetic mice. *Am J Physiol Heart Circ Physiol.* (2011) 301:H306–14. 10.1152/ajpheart.01306.2010 21602470 PMC3154670

[23] Reinehr T Roth C Menke T Andler W. Adiponectin before and after weight loss in obese children. *J Clin Endocrinol Metab.* (2004) 89:3790–4. 10.1210/jc.2003-031925 15292306

[24] Zorena K Jachimowicz-Duda O Ślęzak D Robakowska M Mrugacz M. Adipokines and obesity. Potential link to metabolic disorders and chronic complications. *Int J Mol Sci.* (2020) 21:3570. 10.3390/ijms21103570 32443588 PMC7278967

[25] Varady KA Tussing L Bhutani S Braunschweig CL. Degree of weight loss required to improve adipokine concentrations and decrease fat cell size in severely obese women. *Metabolism.* (2009) 58:1096–101. 10.1016/j.metabol.2009.04.010 19477470

[26] Steppan CM Bailey ST Bhat S Brown EJ Banerjee RR Wright CM The hormone resistin links obesity to diabetes. *Nature.* (2001) 409:307–12. 10.1038/35053000 11201732

[27] Zhang H Wang Y Zhang J Potter BJ Sowers JR Zhang C. Bariatric surgery reduces visceral adipose inflammation and improves endothelial function in type 2 diabetic mice. *Arterioscler Thromb Vasc Biol.* (2011) 31:2063–9. 10.1161/atvbaha.111.225870 21680898 PMC3158262

[28] Chen X Zhang H McAfee S Zhang C. The reciprocal relationship between adiponectin and lox-1 in the regulation of endothelial dysfunction in apoe knockout mice. *Am J Physiol Heart Circ Physiol.* (2010) 299:H605–12. 10.1152/ajpheart.01096.2009 20581092 PMC2944476

[29] Ste Marie L Palmiter RD. Norepinephrine and epinephrine-deficient mice are hyperinsulinemic and have lower blood glucose. *Endocrinology.* (2003) 144:4427–32. 10.1210/en.2003-0561 12959968

[30] Matsuda M Shimomura I Sata M Arita Y Nishida M Maeda N Role of adiponectin in preventing vascular stenosis. the missing link of adipo-vascular axis. *J Biol Chem.* (2002) 277:37487–91. 10.1074/jbc.M206083200 12138120

[31] Su J Lucchesi PA Gonzalez-Villalobos RA Palen DI Rezk BM Suzuki Y Role of advanced glycation end products with oxidative stress in resistance artery dysfunction in type 2 diabetic mice. *Arterioscler Thromb Vasc Biol.* (2008) 28:1432–8. 10.1161/ATVBAHA.108.167205 18483403 PMC2755261

[32] Zhang H Potter BJ Cao JM Zhang C. Interferon-gamma induced adipose tissue inflammation is linked to endothelial dysfunction in type 2 diabetic mice. *Basic Res Cardiol.* (2011) 106:1135–45. 10.1007/s00395-011-0212-x 21826531 PMC3947811

[33] Untergasser A Cutcutache I Koressaar T Ye J Faircloth BC Remm M Primer3–new capabilities and interfaces. *Nucleic Acids Res.* (2012) 40:e115. 10.1093/nar/gks596 22730293 PMC3424584

[34] Zhang H Zhang J Ungvari Z Zhang C. Resveratrol improves endothelial function: role of tnf{alpha} and vascular oxidative stress. *Arterioscler Thromb Vasc Biol.* (2009) 29:1164–71. 10.1161/atvbaha.109.187146 19478208 PMC2761384

[35] Chen X Zhang H Hill MA Zhang C Park Y. Regulation of coronary endothelial function by interactions between Tnf-A, Lox-1 and adiponectin in apolipoprotein e knockout mice. *J Vasc Res.* (2015) 52:372–82. 10.1159/000443887 27050429 PMC5091078

[36] Zhao S Kusminski CM Scherer PE. Adiponectin, leptin and cardiovascular disorders. *Circ Res.* (2021) 128:136–49. 10.1161/circresaha.120.314458 33411633 PMC7799441

[37] Gabel K Kroeger CM Trepanowski JF Hoddy KK Cienfuegos S Kalam F Differential effects of alternate-day fasting versus daily calorie restriction on insulin resistance. *Obesity (Silver Spring).* (2019) 27:1443–50. 10.1002/oby.22564 31328895 PMC7138754

[38] Varady KA Allister CA Roohk DJ Hellerstein MK. Improvements in body fat distribution and circulating adiponectin by alternate-day fasting versus calorie restriction. *J Nutr Biochem.* (2010) 21:188–95. 10.1016/j.jnutbio.2008.11.001 19195863

[39] Wei S Zhao J Bai M Li C Zhang L Chen Y. Comparison of glycemic improvement between intermittent calorie restriction and continuous calorie restriction in diabetic mice. *Nutr Metab (Lond).* (2019) 16:60. 10.1186/s12986-019-0388-x 31485253 PMC6714240

[40] Klempel MC Kroeger CM Norkeviciute E Goslawski M Phillips SA Varady KA. Benefit of a low-fat over high-fat diet on vascular health during alternate day fasting. *Nutr Diabet.* (2013) 3:e71. 10.1038/nutd.2013.14 23712283 PMC3671747

[41] Hoddy KK Bhutani S Phillips SA Varady KA. Effects of different degrees of insulin resistance on endothelial function in obese adults undergoing alternate day fasting. *Nutr Healthy Aging.* (2016) 4:63–71. 10.3233/nha-1611 28035343 PMC5166513

[42] Bhutani S Klempel MC Kroeger CM Trepanowski JF Phillips SA Norkeviciute E Alternate day fasting with or without exercise: effects on endothelial function and adipokines in obese humans. *e-SPEN J.* (2013) 8:e205–9. 10.1016/j.clnme.2013.07.005

[43] Goldstein BJ Scalia R. Adiponectin: a novel adipokine linking adipocytes and vascular function. *J Clin Endocrinol Metab.* (2004) 89:2563–8. 10.1210/jc.2004-0518 15181024

[44] Kubota N Terauchi Y Yamauchi T Kubota T Moroi M Matsui J Disruption of adiponectin causes insulin resistance and neointimal formation. *J Biol Chem.* (2002) 277:25863–6. 10.1074/jbc.C200251200 12032136

[45] Okamoto Y Kihara S Ouchi N Nishida M Arita Y Kumada M Adiponectin reduces atherosclerosis in apolipoprotein e-deficient mice. *Circulation.* (2002) 106:2767–70. 10.1161/01.cir.0000042707.50032.19 12451000

